# Breakfast Consumption in the UK: Patterns, Nutrient Intake and Diet Quality. A Study from the International Breakfast Research Initiative Group

**DOI:** 10.3390/nu10080999

**Published:** 2018-07-30

**Authors:** Szilvia Gaal, Maeve A. Kerr, Mary Ward, Helene McNulty, M. Barbara E. Livingstone

**Affiliations:** Nutrition Innovation Centre for Food and Health (NICHE), Ulster University, Cromore Road, Coleraine BT52 1SA, Northern Ireland, UK; ma.kerr@ulster.ac.uk (M.A.K.); mw.ward@ulster.ac.uk (M.W.); h.mcnulty@ulster.ac.uk (H.M.); mbe.livingstone@ulster.ac.uk (M.B.E.L.)

**Keywords:** breakfast consumption, nutrient intake, diet quality, Nutrient Rich Food Index 9.3, food groups, NDNS

## Abstract

Breakfast consumption is associated with higher overall dietary adequacy; however, there is a lack of quantitative guidelines for optimal nutrient intakes at breakfast in the UK. This study aimed to investigate nutrient and food group intakes at breakfast and examine their relationship to overall Diet Quality (DQ). Data from the most recent National Diet and Nutrition Survey (NDNS, 2008–2014) were accessed to provide a representative sample (*n* = 8174) of the UK population, aged 5–96 years, mean age of 33 years. Food intake was measured by a 4-day estimated food diary and DQ was assessed by the Nutrient Rich Food Index 9.3 method. Energy- and socio-economic-adjusted nutrient and food group intakes were compared across age groups and DQ tertiles by ANCOVA. Breakfast contributed 20–22% to total energy intake. Breakfast intakes of carbohydrate and non-milk extrinsic sugars (NMES) were higher, and intakes of protein, total fat and saturated fatty acid (SFA) were lower, than relative daily intakes. Breakfast was particularly rich in B vitamins, vitamin D, calcium, iron, iodine and magnesium. From the lowest to the highest DQ tertile decreasing intakes of NMES, SFA and total fat and increasing intakes of carbohydrate, protein, fibre and most micronutrients were found. These findings could help to inform the development of nutrient-based recommendations for a balanced breakfast for the first time in the UK.

## 1. Introduction

Evidence suggests that regular breakfast consumption is associated with lower body mass index (BMI) [1,2,3,4], cardiovascular risk [5,6,7,8,9] and enhanced cognitive function [10]. However, although the UK government has been actively promoting breakfast consumption as part of a balanced diet through the “Change4Life” campaign [11], it is estimated that 6% of children aged 7–10 years, 15–26% of adolescents aged 11–15 years and 31% of adults regularly skip breakfast [11]. 

Dietary recommendations in the UK suggest that a healthy breakfast should contribute around 20–25% to total daily energy and nutrient intake [12] and foods should be selected from five main food groups, namely: Starchy foods (cereals, pasta, bread), fruit and vegetables, milk and dairy, protein sources and low-fat spreads and oils [12]. In children aged 5–18 years, the Caroline Walker Trust recommends that breakfast energy and macronutrient contribution should be 20% of the estimated average requirement (EAR) and food energy respectively and that the contribution of breakfast to overall micronutrient intake should be 20% of the Reference Nutrient Intake (RNI) [13]. However, these guidelines are based on the Eatwell Guide and not on actual breakfast food and nutrient intakes of the population [14]. While regular breakfast consumers are reported to have higher nutrient intakes [2,15,16,17,18], and better overall dietary adequacy [15,18] and quality [16,19,20] than non-regular consumers, there is no quantitative nutrient guidance in relation to an appropriate breakfast composition. 

Among UK adults, a cereal based breakfast has been associated with significantly lower intakes of total fat, saturated fatty acid (SFA) and non-milk extrinsic sugars (NMES) and higher intakes of protein, carbohydrate, fibre, B vitamins, vitamin C, D, calcium and iron in comparison to a non-cereal based breakfast or no breakfast [21]. Among UK children (aged 4–10 years) and adolescents (aged 11–18 years), regular breakfast consumers are reported to have significantly higher energy adjusted intakes of dietary fibre, vitamin C, folate, calcium, iron and iodine in comparison to breakfast skippers of the same age [22]. Furthermore, higher total dietary adequacy, in terms of meeting the RNI for vitamin C, folate, calcium, iron and iodine is more readily achieved among children who report regular breakfast consumption compared with children who report breakfast skipping [22]. 

One of the major limitations in generating quantitative recommendations for breakfast has been the lack of consensus on how breakfast should be defined [14]. Previous UK studies investigating breakfast consumption based on National Diet and Nutrition Survey data applied an arbitrary time frame for any item consumed between 6 a.m.–10 a.m. [21] and 6 a.m.–8:59 a.m. with a 100 kcal cut off [22]. Other time frames, energy contribution values and inclusion/exclusion of specific food and beverage groups have also been used as breakfast definitions [23]. A recent definition by O’Neil et al. [23] has been proposed as the basis for providing a consistent approach in future studies: “*Breakfast is the first meal of the day that breaks the fast after the longest period of sleep and is consumed within 2 to 3 h of waking*; *it is comprised of food or beverage from at least one food group*, *and may be consumed at any location*” [23]. The application of the above definition however could lead to inaccurate profiling of breakfast intakes due to differences in dietary survey methodologies [14]. In the UK National Diet and Nutrition Survey (NDNS), breakfast is not pre-defined in the food diary, nor self-defined among NDNS participants. In addition, there is no report of waking time.

The International Breakfast Research Initiative (IBRI) was established to provide evidence based dietary guidelines for nutrient intakes at breakfast by assessing breakfast consumption in representative dietary surveys of the following six countries: Canada, Denmark, France, Spain, UK and the US. The objective of the current paper was to present breakfast consumption patterns, nutrient and food group intakes at breakfast and their contribution to daily intakes in a representative sample of the UK population. Breakfast was defined as any food or beverage item consumed between 6 a.m.–11 a.m. to accommodate different consumption patterns between weekdays and weekend days. The relationship between nutrient and food group intakes at breakfast and overall diet quality using the Nutrient Rich Food Index 9.3 scoring method is also examined according to age.

## 2. Methods

### 2.1. Survey Sample

The National Diet and Nutrition Survey Rolling Programme (2008–2014) is a cross-sectional survey designed to collect information on nutrient intake, food consumption and nutritional status of a representative sample of the UK population living in private households above the age of ≥1.5 years. The full methodology of the survey has been described in detail elsewhere [24]. In brief, survey participants are recruited by a random sampling method based on selection of addresses from Primary Sampling Units (PSUs) of the Postcode Address File. One adult and one child are selected randomly from each household at each address. At the first stage of the survey, interviewers gather information on dietary intake, anthropometric measurements, socio-economic status and physical activity. During the second stage, blood and urine samples are collected by a nurse. Ethical approval for the survey (2008–2012) was provided by the Oxfordshire A Research Ethics Committee [24] and the Cambridge South NRES Committee (Ref. No. 13/EE/0016) for the years between 2012–2014 [25]. The survey and data files were accessed from the UK Data Archive [26]. The total NDNS sample size comprised of 9374 participants between 2008–2014. Breakfast consumption patterns of 8174 participants were analysed in the current study, following the exclusion of children below the age of <5 years (*n* = 1090) and participants who did not consume food and drink items between 6 a.m.–11 a.m. (*n* = 110). Participants were categorised into four age groups: children (5–12 years, *n* = 1947), adolescents (13–18 years, *n* = 1534), adults (19–64 years, *n* = 3619) and older adults (65+ years, *n* = 1074). Non-breakfast consumers (*n* = 110) were only included in the analysis of regularity of breakfast consumption. 

### 2.2. Dietary Assessment in NDNS

Dietary intake data were collected at the first stage of the survey by interviewers during home visits. A 4-day estimated food diary was used to measure food intake on randomly allocated consecutive days including weekdays and weekend days [24]. Household measures, weights from labels and photographs were used to aid the estimation of portion sizes. Food diaries for children below the age of <11 years were completed by a parent or guardian. Food diary checks were carried out by interviewers to assist compliance with completion of food diaries. In order to estimate average energy, macro- and micronutrient intakes, the dietary data were analysed by the Diet In Nutrients Out (DINO) assessment system using food composition data from the Department of Health (DH) NDNS Nutrient Databank [24]. Of the total sample size in this study (*n* = 8174), a four-day food diary was completed by 98.2% participants, while the remainder participants completed three-day food diaries.

### 2.3. Dietary Intake at Breakfast 

As meal occasions are not specified in the NDNS, for the purposes of the current study breakfast was defined as all food and drink items consumed between 6 a.m.–11 a.m. including both weekdays and weekend days. The time frame was determined based on the highest reported number of eating occasions taking place for the four days combined between the hours of midnight and midday. Regularity of breakfast consumption was defined as follows: non-breakfast consumers (*n* = 110) who did not consume any food and drink items between 6 a.m.–11 a.m.; irregular breakfast consumers (*n* = 503) who consumed breakfast on 1 or 2 days out of 4 and regular breakfast consumers (*n* = 7671) who consumed breakfast on 3 or 4 days out of 4. Average energy, macro- and micronutrient intakes at breakfast were calculated based on breakfast consumption days only for the total population and for the four separate age groups. Dietary supplements were excluded from the current analysis in order to assess nutrient intakes from food alone. Mean breakfast energy intake was below 209 kJ (50 kcal) for 142 breakfast consumers, but as the mean ± standard deviation (SD) population energy intake (1442 ± 721 kJ) at breakfast was not significantly different after the exclusion of those participants below 209 kJ (1466 ± 705 kJ), they were retained in all subsequent analyses. Macronutrients included in the analyses were: total carbohydrate, total sugars, NMES, protein, total fat, SFA and fibre (Englyst method [27]). Micronutrients included were: vitamin A, B1, B2, B3, B6, B12, C, D, E, folate, calcium, iodine, iron, potassium, magnesium, sodium and zinc. Englyst fibre was converted to the American Association of Analytical Chemists (AOAC) method by the conversion ratio of 1.33 as outlined by the Scientific Advisory Committee on Nutrition (SACN) [28]. High-fibre breakfast cereals were defined in NDNS as all breakfast cereals with non-starch polysaccharide of ≥4 g/100 g, and other breakfast cereals were defined as all breakfast cereals with non-starch polysaccharide of ≤4 g/100 g. The 20 food groups most frequently consumed between 6 a.m.–11 a.m. by the total population were chosen to calculate the percentage contribution of food groups to key nutrient intakes at breakfast. The same 20 food groups were compared across diet quality tertiles in children and adults. 

### 2.4. Diet Quality Assessment

The Nutrient Rich Food Index (NRF) 9.3 is a validated nutrient profiling method which has been described previously [29]. In the current study, the NRF index was applied to assess overall diet quality (DQ) score in children and adolescents (*n* = 3283) and adults and older adults (*n* = 4891). The algorithm for the index subtracts the sum of three “disqualifying” nutrients (SFA, NMES and salt) from that of nine “qualifying” nutrients (protein, fibre, vitamin A, C, D, calcium, iron, potassium, magnesium) expressed as a multiple of 100.

(∑ sub-scores positive × 100) − (∑ sub-scores negative × 100)

For each nutrient the sub-score was calculated as follows:

((daily intake/daily energy intake) × 8.4 MJ/2000 kcal)/Nutrient Reference Value

If the positive sub-score was higher than 1 then the value of 1 was assigned as sub-score for that nutrient. For each negative nutrient if the sub-score was less than 1 then 0 was assigned to the sub-score, in all other cases 1 was subtracted from the sub-score:

Sub-score = sub-score − 1

The Nutrient Reference Values (NRVs) as defined by the European Parliament [30] were applied in the calculations of qualifying and disqualifying nutrient sub-scores. The upper limit of 10% of energy intake from NMES was used as NRV according to the recommendation by the World Health Organisation (WHO) [31]. Daily sodium intakes were converted to salt by applying the conversion factor of 2.5 [30] and the upper limit of salt was 6 g. The NRV for fibre was defined as 25 g based on EFSA recommendation [32]. 

Tertiles (mean ± SD) of the overall diet quality score were calculated separately for children (*n* = 3283) (T1: 483 ± 68; T2: 608 ± 26; T3: 717 ± 49) and adults (*n* = 4891) (T1: 523 ± 86; T2: 672 ± 30; T3: 788 ± 44) (Tertile 1: low DQ Tertile 2: medium DQ Tertile 3: high DQ). 

### 2.5. Other Variables

Socio-economic status was determined by the National Statistics Socio-Economic Classification (NS-SEC) [33] and survey participants were classified into main groups by housing tenure, education and qualification categories based on the employment of the Household Reference Person (HRP). The derived housing, employment and education variables were recoded further within the data set into the following groups: Housing (own, rent, not applicable); Employment (professional, non-professional, never worked, other); Education (degree, A level, GCSE, foreign, no qualification, still in full time education, not applicable).

Ethnicity was classified into five main categories: White, Mixed (White and Black Caribbean, Black African, Asian), Black or Black British (Caribbean, African), Asian or Asian British (Indian, Pakistani, Bangladeshi) and any ‘other’ ethnic group. 

### 2.6. Statistical Analysis

Normality was assessed by histograms, Q-Q plots, skewness and kurtosis values and Kolmogorov-Smirnov statistic for nutrient and food group intakes and overall NRF 9.3 DQ score. Due to the skewness of the data, both breakfast and daily nutrient intakes were square root transformed, and food groups consumed at breakfast were log10 transformed. All the analyses conducted in the current study followed a harmonized approach defined within the International Breakfast Research Consortium [14]. Regularity of breakfast consumption was compared between gender and age groups by Chi Square test for independence. Descriptive statistics for nutrient and food group intakes are shown as means and standard deviation (SD) of absolute values. Statistical comparison of nutrient intakes between age groups was carried out by ANCOVA adjusted for breakfast and total daily energy intake. One-way ANOVA and ANCOVA adjusted for National Statistics Socio-Economic Classification were applied to compare nutrient and food group intakes across DQ tertiles in children and adults respectively. Mean NRF 9.3 scores were calculated separately for children and adults according to ethnicity and SES (housing, employment, education). However, due to unequal numbers within the subgroups, statistical comparisons could not be carried out. For all analysis, *p* value at ≤0.05 was considered statistically significant and analyses were carried out by SPSS (Version 24) software (SPSS, Chicago, IL, USA). 

## 3. Results

### 3.1. Regularity of Breakfast Consumption

**Figure 1:**
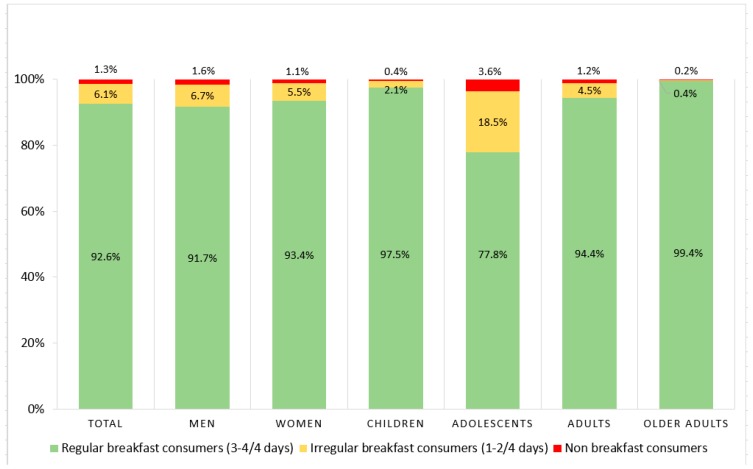
Regularity of breakfast consumption in the total population and stratified by gender and age.

Overall, the majority of the participants (92.6%) were regular breakfast consumers while the remainder were either irregular (6.1%) or non-consumers (1.3%) (Figure 1). A slightly higher proportion of women (93.4%) were regular breakfast consumers compared with men (91.7%) (*p* = 0.011). After stratifying by age, older adults, (65–96 years) (99.4%) consumed breakfast most frequently, followed by children (5–12 years) (97.5%), adults (19–64 years) (94.4%) and adolescents (13–18 years) (77.8%) (*p* = <0.001). The highest proportions of irregular and non-breakfast consumers were adolescents (18.5%, 3.6%), respectively.

### 3.2. Mean Energy, Macronutrient and Micronutrient Intakes Expressed as % Contribution to Breakfast and Daily Energy Intake

Breakfast and daily energy and macronutrient intakes expressed as % contribution to breakfast and daily energy intake respectively were significantly different (*p* < 0.001) across age groups with the exception of total fat intake at breakfast (*p* = 0.104) (Table 1). The highest breakfast and daily energy intakes were observed in adolescents. Higher breakfast intakes of carbohydrate, total sugars and NMES were observed in the total population relative to the daily intakes of these macronutrients. In contrast, breakfast intakes of protein, total fat and SFA were correspondingly lower than daily intakes. The same consistent trend was observed across age groups. At breakfast, adolescents had the highest and older adults had the lowest intake of NMES. In the total population and across age groups mean breakfast AOAC fibre intake was 3 g. Lowest mean daily fibre intake (15 g) was observed in children, followed by adolescents (16 g), adults and older adults (18 g).

Breakfast and total daily micronutrient intakes were significantly different across age groups (Table 2). Older adults had the highest vitamin A intake at breakfast and for the total day (*p* < 0.001). Adolescents had the lowest intake of vitamin A, B12, iodine, potassium and magnesium at breakfast in comparison to the other age groups. However, daily intakes of vitamin D, iron, potassium and magnesium were lower than the reference nutrient intake (RNI) in all age groups. Daily sodium intakes exceeded the recommended 1600 mg in all age groups, with the highest intake being observed in adolescents.

### 3.3. Percentage Contribution of Breakfast to Daily Energy and Nutrient Intakes

**Figure 2:**
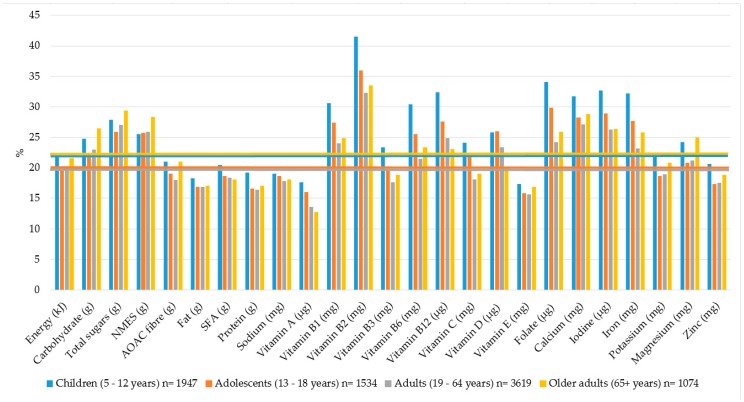
Contribution (%) of breakfast to daily nutrient intakes by age group. Horizontal lines represent the percentage energy contribution from breakfast to total daily energy intake.

Breakfast contributed 20% to daily energy intakes in adolescents and adults and 22% in children and older adults (Figure 2). Total carbohydrate, total sugars and NMES intakes at breakfast contributed proportionately more than 20% to total daily intakes than contribution from total fat, SFA and protein. The contribution of AOAC breakfast fibre to daily intakes was highest in children and older adults (21%), compared with 19% contribution in adolescents and 18% in adults. The contribution of breakfast intakes of vitamin B1, B2, B6, B12, folate, vitamin D, calcium, iron and iodine were the highest to total intakes in all age groups. Overall, children consumed a more micronutrient dense breakfast compared to other age groups. Lower contributions of vitamin A, D and B12 were observed for older adults compared to younger adults. 

### 3.4. Percentage Contribution of Food Groups to Energy and Nutrient Intakes at Breakfast

The contribution of the top 20 food groups most frequently consumed between 6 a.m.–11 a.m. by the total population were assessed to energy and nutrient intakes at breakfast separately for children and adolescents (5–17 years) and adults and older adults (18–96 years). The food groups with the highest percentage of consumers in both age categories were: Tea, coffee, water and semi skimmed milk (Tables S1 and S2). While the younger age category had a high consumption of “other” breakfast cereals, white bread and sugar preserves, sweet spreads, the older age group consumed more sugar preserves, sweet spreads, high-fibre breakfast cereals and fruit at breakfast. The food groups which contributed most to energy intake in both age categories were “other” breakfast cereals, high-fibre breakfast cereals, white bread and semi skimmed milk. Sugar preserves, sweet spreads and “other” breakfast cereals provided 17.7% and 17.3% respectively of NMES intake. Semi skimmed milk and whole milk contributed the most to SFA intake while high-fibre breakfast cereals, white bread and “other” breakfast cereals were the highest contributors to AOAC fibre intake. Micronutrient intakes at breakfast were mainly achieved through the consumption of high-fibre breakfast cereals, “other” breakfast cereals, semi skimmed milk, whole milk, white bread and reduced fat spread in children and adolescents. High-fibre breakfast cereals, “other” breakfast cereals, sugar preserves and sweet spreads, semi skimmed milk and white bread were the highest contributors to both macro- and micronutrient intakes in adults and older adults. 

### 3.5. Nutrient Intakes at Breakfast across Tertiles of NRF9.3 Diet Quality Score

As expected, the mean NRF 9.3 score significantly increased from the lowest quality tertile (T1) to the highest (T3) in both children and adults (*p* < 0.001) (Table 3). Breakfast energy intake did not differ by tertile of DQ score in either of the younger or older age groups. Intakes of NMES, total fat and SFA expressed as % breakfast energy intake significantly decreased from the lowest to the highest DQ tertile (*p* < 0.001) in both children and adults. In contrast, protein and AOAC fibre intake at breakfast showed the opposite trend in both age categories (*p* < 0.001; *p* < 0.001 respectively). 

Micronutrient intakes at breakfast also significantly increased with increasing DQ score in both children and adults (Table 4), particularly intakes of folate, calcium, iron, iodine, potassium and magnesium. In addition, highly significant increases in vitamin A in children (*p* = 0.001) and vitamin C in adults (*p* < 0.001) at breakfast were observed. SES adjusted breakfast sodium intakes decreased from the lowest to the highest DQ tertile in both age categories (*p* = 0.001; *p* < 0.001, respectively).

### 3.6. Food Intakes at Breakfast by Tertiles of NRF 9.3 Diet Quality Score

In children and adolescents, SES-adjusted intakes of semi skimmed milk, high-fibre breakfast cereals and wholemeal bread significantly increased with increasing DQ tertile (*p* < 0.001; *p* = 0.001; *p* = 0.005 respectively) (Table 5). In contrast, adjusted intakes of sugar preserves, sweet spreads, white bread, biscuits, butter and soft drinks (not low energy) showed the opposite trend (*p* = 0.001; *p* = 0.036; *p* = 0.008; *p* = 0.003; *p* = 0.001). The lowest intakes of fruit juice and buns, cakes, pastries and fruit pies were observed in the 3rd tertile, whereas the lowest intake of the miscellaneous food group was observed in the 2nd DQ tertile. Intakes of all other food groups did not significantly differ across DQ tertiles in children and adolescents. 

In adults, intakes of tea coffee and water (*p* < 0.001), semi skimmed milk (*p* < 0.001) and fruit (*p* <0.001) significantly increased from the lowest to the highest tertile of DQ score (Table 6). The opposite was observed for intakes of sugar preserves, sweet spreads, biscuits, butter, bacon and ham, reduced-fat spread, miscellaneous, buns, cakes, pastries and fruit pies, and soft drinks (low- and not-low energy). White bread intake decreased between the lowest and the highest tertile, but not significantly so after adjustment for SES (*p* = 0.078). Higher intake of other breakfast cereals was observed in the 2nd DQ tertile (9.9 g) in comparison to intakes in the 1st and 3rd tertiles (9.4 g). The lowest fruit juice intake was found in the 1st DQ tertile and marginally higher intake in the 2nd tertile than in the 3rd. Intakes of all other foods groups did not significantly differ across DQ tertiles in adults. 

In the lowest DQ tertile children and adults consumed more white bread, sugar preserves and sweet spreads, biscuits, butter, bacon and ham, buns cakes pastries and fruit pies and not low energy soft drinks. In contrast, a higher proportion of children and adults consumed high-fibre breakfast cereals, tea coffee and water, semi skimmed milk, fruit and fruit juice, brown granary, wheatgerm and wholemeal bread and “other” breakfast cereals in the highest DQ tertile. 

## 4. Discussion

The collaborative International Breakfast Research Initiative (IBRI) aims to provide evidence based dietary guidelines for nutrient intakes at breakfast based on nationally representative dietary surveys across six countries: Canada, Denmark, France, Spain, UK and US. This paper presents breakfast consumption patterns, nutrient and food group intakes at breakfast and their contribution to daily intakes in a representative sample of the UK population. The relationship between nutrient and food group intakes at breakfast and overall diet quality are also examined according to age. 

Although the aim of the IBRI collaboration was to apply a harmonised approach to defining breakfast, it was evident that the application of a standard breakfast definition proposed by O’Neil et al. [23] to the UK data presented a particular challenge and it would not fully account for breakfast eating behaviour in the UK. Specifically, breakfast was not defined in the food diary or self-defined by the NDNS participants and no information was provided on waking time. Furthermore, out of the first reported NDNS eating occasions each day (from midnight to midnight), 2.7% took place between midnight and 6 a.m. and 3.3% took place between midday and 11 pm, time frames which may not necessarily include consumption of a typical breakfast meal. Based on an iterative process it was concluded in the current study that the time window of 6 a.m. and 11 a.m. including weekdays and weekend days would best capture breakfast eating behaviour in the UK. 

The present study has shown that the majority of the UK population (92.6%) are regular breakfast consumers, consuming breakfast on at least 3 of the 4 days examined. The prevalence of irregular breakfast consumption, defined as consumption of breakfast on 2 or less days, was found to be highest among adolescents aged 13–18 years (18.5%). This is consistent with findings from previous studies conducted among adolescents in the UK and elsewhere [22,34,35] with differences in family structures, ethnicity [36], lower socioeconomic status [37,38,39], time constraints [40,41] and lack of enjoyment of food in the morning [41] reported to play a role in these trends. 

The key findings of the present study are that breakfast contributed 20–22% to total daily energy intake across all age groups which is consistent with existing recommendations [23]. Notably, however, breakfast contributed marginally more to daily intake of carbohydrate, total sugars and NMES (22–29%) and less to total daily intake of protein, total fat, SFA and dietary fibre (16–21%) compared to the recommended 20–25% by the BDA and the Caroline Walker Trust [12,13]. The recent UK “Sugar reduction programme” aims to reduce total sugar intake and where possible energy without the increase of SFA and salt from the highest contributing food groups by 20% by 2020 [42]. In the first year of the programme, the sugar reduction target of 5% has been largely achieved through reformulation of foods commonly consumed at breakfast such as breakfast cereals [42]. In addition, soft drink reformulation is promoted by the Soft Drinks Industry Levy (SDIL), which took effect in April 2018 and aims to tax soft drinks containing 5–8 g of sugar per 100 mL [42]. The longer-term consequence of the reformulation of foods and drinks typically consumed at breakfast remain to be established. 

Opinions vary as to the optimal macronutrient composition of breakfast [43]. Some studies suggest that a high Glycemic Index (GI)/Glycemic Load (GL) breakfast may have a beneficial effect on cognitive function in participants with normal glucose metabolism, whereas a low-GI/GL breakfast may be more effective on cognition in participants with impaired glucose metabolism [44]. On the other hand, total carbohydrate intake in the highest quintile in comparison to the lowest is reported to increase type 2 diabetes risk whereas partial replacement with dietary fibre may have more favourable metabolic health outcomes [43]. Evidence shows no significant difference in appetite and ad libitum energy intake between a high protein (58.1% protein, 14.1% carbohydrate) versus a high-carbohydrate (19.3% protein, 47.3% carbohydrate) breakfast, although a high-protein breakfast was more effective at reducing postprandial ghrelin concentrations in healthy men [45]. Further recent research has shown that a high-fat breakfast (35% carbohydrate, 20% protein, 45% fat) may be more effective at reducing metabolic disease risk in healthy adults compared to a high-carbohydrate breakfast (60% carbohydrate, 20% protein, 20% fat) [46]. These findings suggest that breakfast compositions higher in protein, fat and fibre and lower in carbohydrate may have a more favourable effect on metabolic disease risk. 

Current available UK-based breakfast recommendations suggest that breakfast should contribute 20% to daily micronutrient intakes [13]. In the present study, the contribution of breakfast to total daily intake of B vitamins, vitamin D, calcium, iodine and iron ranged from 20–41% across all age groups. Thus, breakfast is a particularly nutrient-dense meal in the UK. Previous studies have also observed higher micronutrient intakes [2,15,16,17,18] and improved overall dietary adequacy [15,18] in breakfast consumers. This was found to be mostly the case for children aged 5–12 years in the present study, with lower contributions noted with increasing age. Age differences in breakfast micronutrient intakes were also observed in a previous UK study where children aged 4–10 years had higher intakes of fibre, folate, vitamin C and iodine on breakfast consuming days, whereas no significant differences in intakes were observed among adolescents aged 11–18 years [22]. These findings highlight the importance of maintaining consumption patterns and nutrient intakes from childhood into adolescent age and beyond, especially as eating habits established during childhood tend to track into adulthood [47].

The higher micronutrient intakes at breakfast observed in the current study are likely to be at least partially driven by current UK fortification practices. For example, white bread is mandatorily fortified with vitamin B1, B3, iron and calcium [48]. Furthermore, breakfast cereals, dairy products and fat spreads are fortified on a voluntary basis with vitamin A, D, C, B12 and folate [48]. Consequently, high-fibre and other breakfast cereals, milk, white bread and reduced fat spread were the highest contributors to micronutrient intakes, as well as to energy and fibre. Similarly, ready-to-eat breakfast cereal consumption has been associated with higher fibre and micronutrient intake in other population groups including older American adults [17], Australian children and adolescents [49], low-income UK population [50], Spanish children and adolescents [16] and Black adolescents [51]. Notably, fortified breakfast cereal consumption has been associated with higher dietary adequacy, specifically in relation to the B vitamins, vitamin D and iron, without the risk of exceeding the Tolerable Upper Intake Level intake [52]. 

This study is the first to investigate the relationship between nutrient intakes at breakfast and overall diet quality in the UK by using the Nutrient Rich Food Index 9.3 (NRF 9.3) scoring method and a positive impact of breakfast was observed on overall DQ in both children and adults. The Healthy Eating Index (HEI) and Dietary Diversity Score (DDS) have been used previously to assess the association between breakfast consumption, skippers and diet quality in Iranian women [19], in American adults [53] and American women [54]. It must be noted, however, that in the present study, comparisons of nutrient and food intakes were made based on tertiles of overall DQ in breakfast consumers only; therefore, our results cannot be compared with findings of previous studies which differentiated between breakfast consumers and breakfast skippers [19,53,54]. Nevertheless, higher DQ in the current study corresponded to higher carbohydrate, protein and fibre intakes and lower NMES, total fat and SFA intakes at breakfast. Furthermore, intakes of folate, calcium, iodine, iron, potassium and magnesium and lower intake of sodium at breakfast were observed in the highest DQ tertile in both children and adults. It is also important to note however that actual mean intakes particularly of fibre, calcium, potassium and magnesium differed between children and adults, suggesting the need for age-specific dietary guidelines at breakfast. 

Significant differences were observed in food (g) and beverage (mL) group intakes at breakfast across tertiles of DQ at the total population level; however, intakes were similar across the DQ tertiles in consumers only. These results suggest that there is a tendency towards the choice and consumption of the same food groups. Importantly, also, food group intakes observed in the current study within the highest DQ tertile are consistent with existing UK breakfast recommendations, which suggests the selection of breakfast cereals, wholegrain varieties, semi skimmed milk, fruit, low fat spreads, egg dishes and the limitation of fizzy drinks, biscuits and crisps at breakfast [12]. Future studies could consider the development of coding systems [55,56] and the use of Principal Component Analysis (PCA) to identify key food patterns in relation to nutrient intakes [55,56], which may help to inform the evidence base. 

There are various limitations to the current study which must be acknowledged. Firstly, the cross-sectional design of the study only provides data on prevalence and trends of breakfast patterns in the UK population and a direct link between breakfast intake and dietary adequacy cannot be confirmed. In addition, the breakfast definition used in the current study included both weekdays and weekend days and bias cannot be ruled out due to different associated consumption patterns. Furthermore, weekend days were overrepresented in the NDNS between 2008–2012 [24]. It is also important to note that the contribution of dietary supplements was excluded from the data analysis; however, supplement users, who generally have higher dietary intakes of micronutrients regardless of supplementation [24], were retained. Finally, misreporting of food intake cannot be ruled out in dietary surveys. Exclusion of either under- or over-reporters could, however, have introduced selection and unknown bias [57], for the purpose of the current study, therefore, implausible reporters were not excluded; instead, energy intake was controlled for in statistical analysis. 

In conclusion, the current study has observed that the majority of the UK population are regular breakfast consumers, and breakfast typically contributes 20–22% to total daily energy intake across all age groups. Furthermore, intakes of carbohydrate and NMES at breakfast are typically higher and intakes of protein, total fat and SFA are typically lower at breakfast than for the total day in this cohort. The current study has also provided evidence that the UK breakfast is a micronutrient rich meal across all age groups, impacting particularly on daily intakes of key nutrients including vitamin D, calcium and folate. The results from this study also provide strong evidence for a positive impact of breakfast consumption on overall diet quality. These findings could help to inform the development of nutrient based recommendations for a balanced breakfast for the first time in the UK.

## Figures and Tables

**Table 1:** Mean energy and macronutrient intakes at breakfast and for the total day in the total population and by age group.

	Total Population (*n* = 8174)				Children 5–12 years (*n* = 1947)				Adolescents 13–18 years (*n* = 1534)				Adults 19–64 years (*n* = 3619)				Older Adults 65+ Years (*n* = 1074)				*p* Value *	*p* Value **
	Breakfast Intake		Daily Intake		Breakfast Intake		Daily Intake		Breakfast Intake		Daily Intake		Breakfast Intake		Daily Intake		Breakfast Intake		Daily Intake		Breakfast Intake	Daily Intake
	Mean	SD	Mean	SD	Mean	SD	Mean	SD	Mean	SD	Mean	SD	Mean	SD	Mean	SD	Mean	SD	Mean	SD		
Energy (kJ)	1442	721	7062	2049	1450	541	6664	1489	1471	773	7518	2202	1425	795	7193	2223	1444	668	6690	1895	˂0.001	˂0.001
Carbohydrates (g)	52	25	220	68	54	19	218	51	54	27	242	74	50	26	218	72	53	25	201	62	˂0.001	˂0.001
Carbohydrates (% E)	59	13	49	6	60	10	52	5	60	13	51	6	58	14	48	7	59	12	47	6	˂0.001	˂0.001
Total sugars (g)	26	16	96	42	27	13	99	35	26	17	105	47	24	16	93	44	26	16	89	38	˂0.001	˂0.001
Total sugars (% E)	30	15	21	7	30	11	23	6	30	16	22	7	30	16	20	7	30	14	21	6	0.009	˂0.001
NMES (g)	15	13	60	38	16	11	63	30	18	15	76	42	14	12	56	39	14	12	48	30	˂0.001	˂0.001
NMES (% E)	17	14	13	6	17	10	15	6	21	15	16	7	17	15	12	7	15	12	11	5	˂0.001	˂0.001
AOAC fibre (g)	3	2	17	6	3	2	15	4	3	2	16	5	3	3	18	7	4	3	18	6	˂0.001	˂0.001
Fat (g)	11	9	65	24	11	6	59	17	11	9	68	24	11	9	67	26	11	8	63	23	0.007	˂0.001
Fat (% E)	28	11	34	6	27	9	34	5	28	12	34	5	28	12	35	6	27	12	35	6	0.104	˂0.001
SFA (g)	5	4	24	10	5	3	23	8	5	4	25	10	5	4	25	11	4	4	25	10	˂0.001	˂0.001
SFA (% E)	12	6	13	3	12	5	13	3	11	5	13	3	11	6	13	3	11	6	14	4	˂0.001	˂0.001
Protein (g)	12	7	67	22	11	5	58	14	11	8	67	23	12	8	72	23	12	6	68	19	˂0.001	˂0.001
Protein (% E)	14	5	16	4	13	3	15	2	13	5	15	3	14	6	17	4	14	5	17	3	˂0.001	˂0.001

*p* * adjusted for energy intake at breakfast (ANCOVA). *p* ** adjusted for daily energy intake (ANCOVA). SD: standard deviation. Macronutrient values were square root transformed for normalization purposes prior to analysis; data shown as absolute values. Macronutrients at breakfast are expressed as percentage contribution to breakfast energy; daily macronutrient intakes expressed as percentage contribution to daily food energy intake. *p* < 0.05 is considered significant. Abbreviations: NMES non-milk extrinsic sugars; SFA saturated fatty acid; AOAC American Association of Analytical Chemists.

**Table 2:** Mean micronutrient intakes at breakfast and for the total day in the total population and by age group.

	Total Population (*n* = 8174)				Children 5–12 years (*n* = 1947)				Adolescents 13–18 years (*n* = 1534)				Adults 19–64 years (*n* = 3619)				Older Adults 65+ Years (*n* = 1074)				*p* Value *	*p* Value **
	Breakfast Intake		Daily Intake		Breakfast Intake		Daily Intake		Breakfast Intake		Daily Intake		Breakfast Intake		Daily Intake		Breakfast Intake		Daily Intake		Breakfast Intake	Daily Intake
	Mean	SD	Mean	SD	Mean	SD	Mean	SD	Mean	SD	Mean	SD	Mean	SD	Mean	SD	Mean	SD	Mean	SD		
Vitamin A (µg)	100	201	826	844	99	126	627	445	92	212	650	502	102	228	907	872	107	194	1166	1374	˂0.001	˂0.001
Vitamin B1 (mg)	0.4	0.2	1.4	0.5	0.4	0.2	1.3	0.4	0.4	0.3	1.4	0.5	0.4	0.3	1.4	0.5	0.4	0.2	1.4	0.5	˂0.001	˂0.001
Vitamin B2 (mg)	0.6	0.4	1.5	0.6	0.6	0.3	1.5	0.6	0.5	0.4	1.5	0.7	0.5	0.4	1.6	0.7	0.6	0.3	1.6	0.6	˂0.001	˂0.001
Vitamin B3 (mg)	6	4	32	12	6	3	28	8	7	5	33	13	6	5	35	13	6	3	31	10	˂0.001	˂0.001
Vitamin B6 (mg)	0.5	0.4	2.0	1.0	0.5	0.3	1.8	0.7	0.5	0.5	2.1	1.2	0.5	0.4	2.1	1.1	0.5	0.4	2.0	0.8	˂0.001	˂0.001
Vitamin B12 (µg)	1.2	1.1	4.8	3.2	1.3	0.8	4.1	1.8	1.1	1.0	4.2	2.5	1.2	1.3	5.0	3.4	1.2	1.0	6.0	4.5	˂0.001	˂0.001
Vitamin C (mg)	18	24	80	54	22	22	84	49	19	26	78	55	17	25	79	58	17	22	77	48	˂0.001	˂0.001
Vitamin D (µg)	0.6	0.8	2.6	1.8	0.6	0.6	2.0	1.2	0.6	0.8	2.2	1.4	0.7	0.9	2.8	2.0	0.7	0.9	3.3	2.3	0.003	˂0.001
Vitamin E (mg)	1.4	1.9	8.6	3.8	1.3	0.9	7.4	2.6	1.4	1.7	8.9	3.8	1.5	2.4	9.1	4.2	1.4	1.2	8.3	3.5	0.005	˂0.001
Folate (µg)	64	46	227	94	69	39	196	68	65	49	210	86	62	48	246	102	66	47	245	94	˂0.001	˂0.001
Calcium (mg)	229	138	801	307	258	139	808	283	218	145	784	337	217	135	802	310	232	129	811	292	˂0.001	˂0.001
Iodine (µg)	41	30	151	78	45	30	136	64	36	31	127	76	41	28	160	79	46	29	180	86	˂0.001	˂0.001
Iron (mg)	2.7	1.8	9.8	3.4	2.9	1.6	8.8	2.5	2.8	2.0	9.6	3.4	2.5	1.8	10.3	3.6	2.6	1.7	9.9	3.3	˂0.001	˂0.001
Potassium (mg)	506	283	2534	808	491	223	2194	546	435	257	2345	755	525	310	2742	867	573	301	2719	801	˂0.001	˂0.001
Magnesium (mg)	51	31	227	79	48	22	195	50	44	26	211	69	54	34	250	88	60	32	236	77	˂0.001	˂0.001
Sodium (mg)	388	316	2107	756	357	219	1895	551	420	343	2232	778	396	350	2199	822	368	294	2002	719	˂0.001	˂0.001
Zinc (mg)	1.4	0.9	7.7	2.8	1.3	0.7	6.6	2.0	1.3	0.9	7.5	2.7	1.5	1.0	8.3	3.0	1.5	0.8	8.1	2.6	˂0.001	˂0.001

*p* * adjusted for energy intake at breakfast (ANCOVA) *p* ** adjusted for daily energy intake (ANCOVA). Micronutrient values were square root transformed for normalisation purposes prior to analysis; data shown as absolute values. *p* < 0.05 is considered significant.

**Table 3:** Mean (SD) intake of energy and macronutrients at breakfast across tertiles of NRF 9.3 score by age group.

	Children (5–17 years) (*n* = 3283)								Adults (18+ Years) (*n* = 4891)							
	T1 (*n* = 1094)		T2 (*n* = 1095)		T3 (*n* = 1094)				T1 (*n* = 1630)		T2 (*n* = 1631)		T3 (*n* = 1630)			
	Mean	SD	Mean	SD	Mean	SD	*p* Value *	*p* Value **	Mean	SD	Mean	SD	Mean	SD	*p* Value *	*p* Value **
NRF 9.3 score	483	68	608	26	717	49	˂0.001	˂0.001	523	86	672	30	788	44	˂0.001	˂0.001
Energy (kJ) ‡	1518	738	1452	615	1439	575	0.156	0.113	1452	858	1443	756	1375	673	0.204	0.057
Carbohydrates (g) ‡	55	26	53	22	54	21	0.491	0.399	49	27	50	26	52	25	0.001	0.076
Carbohydrates (% E) ‡	59	12	60	11	61	10	0.002	0.003	57	15	57	13	61	12	˂0.001	˂0.001
Total sugars (g) ‡	29	18	26	15	25	13	˂0.001	˂0.001	26	17	23	16	25	15	˂0.001	˂0.001
Total sugars (% E) ‡	32	15	30	13	29	12	˂0.001	˂0.001	33	20	27	14	30	14	˂0.001	˂0.001
NMES (g) ‡	21	16	16	12	13	9	˂0.001	˂0.001	18	15	13	11	11	9	˂0.001	˂0.001
NMES (% E) ‡	23	15	18	12	14	9	˂0.001	˂0.001	23	19	14	12	12	10	˂0.001	˂0.001
AOAC fibre (g) ‡	2.5	1.9	3.0	1.8	3.7	2.2	˂0.001	˂0.001	2.4	2.0	3.5	2.5	4.6	3.0	˂0.001	˂0.001
Fat (g) ‡	12	9	11	7	10	6	˂0.001	˂0.001	13	11	12	9	9	7	˂0.001	˂0.001
Fat (% E) ‡	29	11	28	10	25	10	˂0.001	˂0.001	30	13	29	12	24	10	˂0.001	˂0.001
SFA (g) ‡	5	4	5	3	4	3	˂0.001	˂0.001	6	5	5	4	3	3	˂0.001	˂0.001
SFA (% E) ‡	13	6	12	5	11	4	˂0.001	˂0.001	13	7	12	6	9	4	˂0.001	˂0.001
Protein (g) ‡	11	7	11	6	12	6	˂0.001	˂0.001	11	8	12	8	12	7	˂0.001	˂0.001
Protein (% E) ‡	12	4	13	4	14	4	˂0.001	˂0.001	12	6	14	5	16	5	˂0.001	˂0.001

Tertile 1: Low DQ; Tertile 2: Medium DQ; Tertile 3: High DQ; *p* * unadjusted (ANOVA). *p* ** adjusted for National Statistics Socio-economic Classification (housing, employment, education) (ANCOVA). ‡ Nutrient values were square root transformed for normalization purposes prior to analysis; data shown as absolute values. Macronutrients expressed as percentage contribution to energy intake at breakfast. *p* < 0.05 is considered significant. Abbreviations: NMES non-milk extrinsic sugars; SFA saturated fatty acid; AOAC American Association of Analytical Chemists.

**Table 4:** Mean (SD) intake of micronutrients at breakfast across tertiles of NRF 9.3 score by age group.

	Children (5–17 years) (*n* = 3283)								Adults (18+ Years) (*n* = 4891)							
	T1 (*n* = 1094)		T2 (*n* = 1095)		T3 (*n* = 1094)				T1 (*n* = 1630)		T2 (*n* = 1631)		T3 (*n* = 1630)			
	Mean	SD	Mean	SD	Mean	SD	*p* Value *	*p* Value **	Mean	SD	Mean	SD	Mean	SD	*p* Value *	*p* Value **
Vitamin A (µg)	90	143	96	136	107	227	0.001	0.001	98	135	109	192	97	293	˂0.001	0.001
Vitamin B1 (mg)	0.4	0.2	0.4	0.2	0.4	0.2	˂0.001	˂0.001	0.3	0.2	0.4	0.2	0.4	0.3	˂0.001	˂0.001
Vitamin B2 (mg)	0.5	0.4	0.6	0.3	0.7	0.4	˂0.001	˂0.001	0.4	0.4	0.5	0.4	0.6	0.4	˂0.001	˂0.001
Vitamin B3 (mg)	6	4	6	3	7	4	˂0.001	˂0.001	5	5	6	4	7	4	˂0.001	˂0.001
Vitamin B6 (mg)	0.5	0.5	0.5	0.3	0.6	0.4	˂0.001	˂0.001	0.4	0.5	0.5	0.4	0.5	0.4	˂0.001	˂0.001
Vitamin B12 (µg)	1.1	0.9	1.2	0.9	1.4	1.0	˂0.001	˂0.001	1.1	1.1	1.2	1.0	1.3	1.4	˂0.001	˂0.001
Vitamin C (mg)	18	23	22	24	22	24	˂0.001	˂0.001	11	18	16	24	23	27	˂0.001	˂0.001
Vitamin D (µg)	0.5	0.6	0.6	0.7	0.7	0.8	˂0.001	˂0.001	0.5	0.7	0.7	0.9	0.8	0.9	˂0.001	˂0.001
Vitamin E (mg)	1.3	1.7	1.3	1.0	1.4	1.2	˂0.001	˂0.001	1.2	1.2	1.5	1.4	1.7	3.2	˂0.001	˂0.001
Folate (µg)	56	39	67	40	79	44	˂0.001	˂0.001	47	41	64	52	76	48	˂0.001	˂0.001
Calcium (mg)	226	140	244	145	260	142	˂0.001	˂0.001	201	135	224	137	232	128	˂0.001	˂0.001
Iodine (µg)	35	28	41	29	48	34	˂0.001	˂0.001	36	27	42	29	46	29	˂0.001	˂0.001
Iron (mg)	2.5	1.6	2.9	1.7	3.3	1.8	˂0.001	˂0.001	2.0	1.6	2.5	1.8	3.0	1.8	˂0.001	˂0.001
Potassium (mg)	419	231	470	230	523	243	˂0.001	˂0.001	420	249	535	308	637	322	˂0.001	˂0.001
Magnesium (mg)	41	22	46	23	53	26	˂0.001	˂0.001	41	25	54	32	68	38	˂0.001	˂0.001
Sodium (mg)	420	338	378	263	356	219	˂0.001	0.001	424	398	409	334	337	262	˂0.001	˂0.001
Zinc (mg)	1.2	0.8	1.3	0.7	1.5	0.8	˂0.001	˂0.001	1.3	0.9	1.5	0.9	1.6	1.1	˂0.001	˂0.001

Tertile 1: Low DQ; Tertile 2: Medium DQ; Tertile 3: High DQ. *p* * unadjusted (ANOVA). *p* ** adjusted for National Statistics Socio-economic Classification (housing, employment, education) (ANCOVA). Nutrients were square root transformed for normalization purposes prior to analysis, data shown as absolute values. *p* < 0.05 is considered significant.

**Table 5:** Food intakes and % of consumers across tertiles of NRF 9.3 score in children and adolescents.

	Children and Adolescents (5–17) years (*n* = 3283)										
	T1 (*n* = 1094)			T2 (*n* = 1095)			T3 (*n* = 1094)				
	Mean	SD	% Consumers	Mean	SD	% Consumers	Mean	SD	% Consumers	*p* Value *	*p* Value **
Tea coffee and water (mL)	146.9	165	57	152	168	61	163.5	174	66	0.323	0.541
Semi skimmed milk (mL)	74.4	95	54	90.2	96	64	104.2	102	66	˂0.001	˂0.001
Sugar preserves and sweet spreads (g)	6.6	10	46	6.4	11	46	5.4	10	43	0.001	0.001
High-fibre breakfast cereals (g)	13.7	30	31	22	36	46	28.2	38	57	0.001	0.001
White bread (g)	30.7	36	53	25.2	32	47	22.4	33	40	0.020	0.036
Fruit (g)	23.6	54	23	42	61	42	54.7	63	53	0.327	0.331
Other breakfast cereals (g)	15.9	21	45	16.5	20	49	17.3	21	51	0.225	0.385
Reduced fat spread (g)	3.5	6	28	4	7	34	4	7	34	0.207	0.531
Fruit juice (mL)	60.8	110	28	69.3	105	35	58.6	97	32	0.016	0.025
Biscuits (g)	9.2	19	27	8.1	16	27	6.4	14	23	0.006	0.008
Whole milk (mL)	40.3	83	26	40.3	83	25	41.5	83	25	0.237	0.279
Eggs and egg dishes (g)	8.5	27	12	12	32	16	13	33	17	0.561	0.513
Miscellaneous (g)	4.3	18	19	3.3	18	16	3.6	24	17	0.003	0.006
Butter (g)	2.7	7	18	2.1	6	16	1.5	5	12	0.002	0.003
Bacon and ham (g)	9.0	22	18	6.1	17	14	4.5	15	10	0.152	0.265
Brown granary and wheatgerm bread (g)	5.6	19	9	8	22	15	9.4	23	17	0.172	0.236
Buns, cakes, pastries & fruit pies (g)	11.2	28	17	14	33	21	7.9	22	14	0.023	0.036
Wholemeal bread (g)	3.1	15	5	4.7	16	10	7.9	20	16	0.002	0.005
Soft drinks not low calorie (mL)	105.9	166	36	49.1	115	19	26.1	86	11	˂0.001	0.001
Soft drinks low calorie (mL)	53.4	114	21	59.4	122	23	61	123	24	0.923	0.602

Tertile 1: Low DQ; Tertile 2: Medium DQ; Tertile 3: High DQ. *p* * unadjusted (ANOVA). *p* ** adjusted for National Statistics Socio-economic Classification (housing, employment, education) (ANCOVA). Food groups were Log10 transformed for normalization purposes prior to analysis, data shown as absolute values. No statistical tests were carried out for % of consumers. *p* < 0.05 is considered significant.

**Table 6:** Food intakes and % of consumers across tertiles of NRF 9.3 score in adults.

	Adults (18+ Years) (*n*= 4891)										
	T1 (*n* = 1630)			T2 (*n* = 1631)			T3 (*n* = 1630)				
	Mean	SD	% Consumers	Mean	SD	% Consumers	Mean	SD	% Consumers	*p* Value *	*p* Value **
Tea coffee and water (mL)	366.5	226	92	402.1	217	97	466.5	242	98	˂0.001	˂0.001
Semi skimmed milk (mL)	56.6	73	62	72.1	83	69	76.3	81	69	˂0.001	˂0.001
Sugar preserves and sweet spreads (g)	10.9	12	67	8.1	10	56	6.7	10	47	˂0.001	˂0.001
High-fibre breakfast cereals (g)	15.9	38	26	27.6	49	44	34.9	47	58	0.923	0.818
White bread (g)	30.2	37	50	25.7	34	44	13.4	28	24	0.032	0.078
Fruit (g)	23.1	53	22	43.2	67	42	70	83	61	˂0.001	˂0.001
Other breakfast cereals (g)	9.4	18	26	9.9	18	29	9.4	17	28	0.003	0.002
Reduced fat spread (g)	4.7	8	32	4.6	8	33	3.4	6	28	˂0.001	˂0.001
Fruit juice (mL)	30.5	86	14	41.7	93	21	41.5	87	24	˂0.001	˂0.001
Biscuits (g)	7.2	17	22	7	16	23	5.6	12	22	˂0.001	˂0.001
Whole milk (mL)	28.7	69	27	20.4	61	18	8.2	36	8	0.766	0.853
Eggs and egg dishes (g)	14.4	36	17	16.9	37	21	20.2	41	23	0.525	0.306
Miscellaneous (g)	6.3	33	17	5.1	30	17	4.4	25	20	˂0.001	˂0.001
Butter (g)	4.0	8	24	3.4	7	22	1.4	4	12	˂0.001	˂0.001
Bacon and ham (g)	10.9	25	20	9.9	24	19	5.9	18	12	0.031	0.026
Brown granary and wheatgerm bread (g)	6.9	21	12	9.3	23	17	11	24	20	0.102	0.141
Buns, cakes, pastries & fruit pies (g)	11.5	30	16	8.9	25	14	5.8	19	10	˂0.001	˂0.001
Wholemeal bread (g)	5.7	19	10	9.9	24	18	13.6	27	26	0.157	0.272
Soft drinks not low calorie (mL)	59.1	153	16	20.7	86	7	13.6	65	6	˂0.001	˂0.001
Soft drinks low calorie (mL)	35.2	129	9	24.5	102	6	22.5	96	7	0.021	0.017

Tertile 1: Low DQ; Tertile 2: Medium DQ; Tertile 3: High DQ. *p* * unadjusted (ANOVA) *p* ** adjusted for National Statistics Socio-economic Classification (housing, employment, education) (ANCOVA). Food groups were Log10 transformed for normalization purposes prior to analysis, data shown as absolute values. No statistical tests were carried out for % of consumers. *p* < 0.05 is considered significant.

## Supplementary Materials

The following are available online at http://www.mdpi.com/2072-6643/10/8/999/s1, Table S1: Contribution (%) of food groups to intakes of key nutrients at breakfast in children and adolescents aged 5–17 years, Table S2: Contribution (%) of food groups to intakes of key nutrients at breakfast in adults and older adults aged 18–96 years.
