# Supplementary material for: Breakfast Consumption in the UK: Patterns, Nutrient Intake and Diet Quality. A Study from the International Breakfast Research Initiative Group
Source: Nutrients. 2018 Jul 30;10(8):999. doi: 10.3390/nu10080999 (PMC6115898; doi:10.3390/nu10080999)
Supplement: Supplementary file 1 [file nutrients-10-00999-s001.pdf]

Supplementary Material

Table S1. Contribution (%) of food groups to intakes of key nutrients at breakfast in children and adolescents aged 5-17 years.

	% of consumers	Energy	NMES	AOAC fibre	Saturated fat	Sodium	Vitamin B12	Vitamin D	Folate	Calcium	Iron	Potassium	Magnesium
Tea, coffee and water	62	0.1	0.0	0.0	0.1	0.1	0.1	0.0	0.9	0.1	0.1	1.8	1.2
Semi skimmed milk	61	9.6	0.0	0.0	22.0	10.8	40.0	0.1	8.3	31.0	0.0	22.2	15.1
Other breakfast cereals	48	11.6	17.3	12.9	2.9	12.2	8.0	19.5	21.7	5.7	26.3	4.1	8.2
White bread	47	9.8	0.4	15.8	1.9	15.1	0.0	0.1	8.1	12.0	11.4	5.6	8.7
Sugar preserves and sweet spreads	45	3.8	17.7	0.6	1.4	0.3	0.2	0.0	0.2	0.5	1.3	0.8	1.0
High fibre breakfast cereals	45	11.1	11.1	24.1	3.6	9.4	5.0	7.5	19.2	5.8	26.3	7.6	14.1
Fruit	39	4.0	0.9	11.5	0.6	0.5	0.0	0.0	3.9	1.4	2.6	8.5	6.3
Reduced fat spread	32	3.1	0.0	0.0	8.6	2.9	2.1	21.3	2.3	0.2	0.1	0.3	0.1
Fruit juice	32	4.0	15.7	1.8	0.4	1.0	0.0	0.0	7.2	2.0	2.6	8.8	5.5
Biscuits	25	4.5	6.2	4.3	6.3	3.5	1.1	1.1	2.2	2.5	4.4	2.4	3.4
Whole milk	25	5.5	0.0	0.0	12.9	4.6	15.8	0.0	3.7	12.9	0.0	9.5	6.6
Soft drinks low calorie	23	0.3	1.4	0.0	0.0	1.2	0.3	0.1	0.5	0.5	0.1	0.9	0.6
Soft drinks not low calorie	22	2.8	10.2	0.1	0.0	1.0	0.9	0.0	0.6	1.0	0.3	1.3	1.5
Buns, cakes, pastries & fruit pies	18	3.9	3.7	3.9	4.8	4.5	1.2	5.5	2.3	2.3	3.1	2.0	2.4
Miscellaneous	18	0.9	2.7	0.6	0.7	1.9	1.0	0.9	1.9	0.5	1.3	1.4	1.6
Butter	15	1.9	0.0	0.0	6.7	1.2	0.9	4.9	0.0	0.1	0.0	0.1	0.1
Eggs and egg dishes	15	1.7	0.0	0.0	2.7	1.6	5.5	11.1	2.3	1.2	2.7	1.2	1.1
Bacon and ham	14	1.4	0.2	0.0	2.7	4.4	2.4	4.6	0.1	0.1	0.8	1.8	1.2
Brown granary and wheatgerm bread	14	2.5	0.0	5.5	0.5	3.8	0.0	0.0	2.6	3.7	3.8	2.0	3.7
Wholemeal bread	10	1.7	0.0	3.9	0.4	2.8	0.0	0.0	1.7	1.6	2.4	1.5	2.8
**Total**		84.1	87.7	85.0	79.3	82.7	84.4	76.6	88.8	84.9	89.7	82.1	84.1

Miscellaneous food group includes: Savory sauces, pickles, gravies & condiments; beverages dry weight; Nutrition powders & drinks; Soup manufactured/retail; Soup homemade. Abbreviations: NMES non-milk extrinsic sugars; AOAC American Association of Analytical Chemists.

Table S2. Contribution (%) of food groups to intakes of key nutrients at breakfast in adults and older adults aged 18 years +.

	% of consumers	Energy	NMES	AOAC fibre	Saturated fat	Sodium	Vitamin B12	Vitamin D	Folate	Calcium	Iron	Potassium	Magnesium
**Tea coffee and water**	96	1.0	0.6	0.0	1.0	1.2	1.3	0.1	6.1	2.2	4.3	15.3	11.2
**Semi skimmed milk**	67	9.7	0.0	0.0	21.9	12.4	41.9	0.6	9.6	32.1	0.1	18.5	12.5
**Sugar preserves and sweet spreads**	57	7.5	36.6	0.5	0.2	0.5	0.0	0.1	0.1	0.5	1.9	0.5	0.5
**High fibre breakfast cereals**	42	12.1	11.3	24.1	5.6	9.5	4.4	8.1	16.3	5.9	23.6	8.2	14.9
**Fruit**	42	5.8	2.3	12.1	1.1	0.8	0.1	0.0	5.7	2.0	4.5	10.7	8.0
**White bread**	39	9.1	0.3	16.0	1.7	13.5	0.1	0.3	8.1	11.1	11.9	4.9	7.6
**Reduced fat spread**	31	3.5	0.0	0.0	9.7	3.2	1.9	21.3	2.4	0.2	0.1	0.3	0.1
**Other breakfast cereals**	28	6.6	9.3	7.8	1.6	7.3	4.8	12.3	12.8	1.4	15.0	1.9	3.7
**Biscuits**	23	3.9	6.3	4.5	5.4	3.5	0.4	1.3	1.3	1.7	3.7	1.6	2.5
**Eggs and egg dishes**	20	2.7	0.0	0.0	4.2	2.2	8.2	15.7	3.9	1.8	4.7	1.7	1.7
**Fruit juice**	20	2.4	10.3	1.2	0.2	0.9	0.0	0.0	4.9	1.3	2.1	4.7	3.0
**Butter**	20	2.9	0.0	0.0	9.7	1.8	0.9	7.3	0.0	0.1	0.0	0.1	0.1
**Miscellaneous**	18	1.1	2.4	0.9	1.1	2.8	1.4	1.1	2.3	0.9	1.5	1.5	1.5
**Wholemeal bread**	18	3.6	0.1	8.1	1.0	5.9	0.0	0.0	3.7	3.2	5.5	2.9	5.4
**Whole milk**	18	3.1	0.0	0.0	7.4	3.1	10.6	0.1	2.9	8.3	0.0	5.3	3.7
**Bacon and ham**	17	1.9	0.2	0.0	3.3	5.7	2.3	4.7	0.1	0.1	1.0	2.1	1.4
**Brown granary and wheatgerm bread**	16	3.4	0.1	6.9	0.8	5.0	0.0	0.0	3.9	4.7	5.1	2.3	4.3
**Buns, cakes, pastries & fruit pies**	13	3.0	3.6	3.0	3.6	3.3	0.8	3.4	1.7	1.7	2.7	1.4	1.6
**Soft drinks not low calorie**	10	1.5	5.5	0.0	0.0	0.5	0.5	0.0	0.3	0.5	0.2	0.5	0.7
**Soft drinks low calorie**	8	0.1	0.7	0.0	0.0	0.4	0.1	0.0	0.1	0.4	0.0	0.3	0.3
**Total**		83.7	89.1	85.3	78.6	82.3	78.4	76.3	80.3	78.2	83.7	69.2	73.4

Miscellaneous food group includes: Savory sauces, pickles, gravies & condiments; beverages dry weight; Nutrition powders & drinks; Soup manufactured/retail; Soup homemade. Abbreviations: NMES non-milk extrinsic sugars; AOAC American Association of Analytical Chemists.
